# *In situ* Occurrence, Prevalence and Dynamics of *Parvilucifera* Parasitoids during Recurrent Blooms of the Toxic Dinoflagellate *Alexandrium minutum*

**DOI:** 10.3389/fmicb.2017.01624

**Published:** 2017-08-31

**Authors:** Elisabet Alacid, Albert Reñé, Jordi Camp, Esther Garcés

**Affiliations:** Departament de Biologia Marina i Oceanografia, Institut de Ciències del Mar, CSIC Barcelona, Spain

**Keywords:** phytoplankton, plankton, protist, harmful algal blooms, Perkinsids, parasitism, infection flux

## Abstract

Dinoflagellate blooms are natural phenomena that often occur in coastal areas, which in addition to their large number of nutrient-rich sites are characterized by highly restricted hydrodynamics within bays, marinas, enclosed beaches, and harbors. In these areas, massive proliferations of dinoflagellates have harmful effects on humans and the ecosystem. However, the high cell density reached during blooms make them vulnerable to parasitic infections. Under laboratory conditions parasitoids are able to exterminate an entire host population. In nature, *Parvilucifera* parasitoids infect the toxic dinoflagellate *Alexandrium minutum* during bloom conditions but their prevalence and impact remain unexplored. In this study, we evaluated the *in situ* occurrence, prevalence, and dynamics of *Parvilucifera* parasitoids during recurrent blooms of *A. minutum* in a confined site in the NW Mediterranean Sea as well as the contribution of parasitism to bloom termination. *Parvilucifera* parasitoids were recurrently detected from 2009 to 2013, during seasonal outbreaks of *A. minutum*. Parasitic infections in surface waters occurred after the abundance of *A. minutum* reached 10^4^–10^5^ cells L^−1^, suggesting a density threshold beyond which *Parvilucifera* transmission is enhanced and the number of infected cells increases. Moreover, host and parasitoid abundances were not in phase. Instead, there was a lag between maximum *A. minutum* and *Parvilucifera* densities, indicative of a delayed density-dependent response of the parasitoid to host abundances, similar to the temporal dynamics of predator-prey interactions. The highest parasitoid prevalence was reached after a peak in host abundance and coincided with the decay phase of the bloom, when a maximum of 38% of the *A. minutum* population was infected. According to our estimates, *Parvilucifera* infections accounted for 5–18% of the total observed *A. minutum* mortality, which suggested that the contribution of parasitism to bloom termination is similar to that of other biological factors, such as encystment and grazing.

## Introduction

In the last decades, toxic and harmful planktonic protist species have been the focus of scientific and public attention due to their environmental, economic, and public health impacts in coastal areas, which are of major importance for food production (Zingone and Enevoldsen, 2000). Around 200 species belonging to diverse groups of marine microalgae, including dinoflagellates, diatoms, pelagophytes, raphydophytes, and prymnesiophytes, have been identified as potentially harmful. Of these, about 90 species, mainly those of dinoflagellates, are potentially toxic (Zingone and Enevoldsen, 2000; Hallegraeff et al., 2003).

In the Mediterranean Sea, harmful algal blooms (HABs) commonly occur in areas with restricted hydrodynamics, such as bays, lagoons, harbors, beaches, and estuaries. These coastal proliferations are an emerging problem whose frequency has increased in response to the intensive urbanization and recreational use of the Mediterranean shoreline, which has resulted in nutrient-rich (semi-) confined areas with low turbulence levels. These areas constitute a unique environment that favors HAB formation by several planktonic protist species (Garcés and Camp, 2012). For example, the worldwide distributed *Alexandrium minutum* is responsible for outbreaks of paralytic shellfish poisoning in humans and for the high mortality of wild and cultured aquatic fauna (Anderson et al., 2012). It also forms recurrent blooms along the Catalan coast (NW Mediterranean Sea) (Vila et al., 2001, 2005; Bravo et al., 2008), which includes a large number of harbors and suffers from huge nutrient inputs from inland sources (Vila et al., 2001; Garcés et al., 2003; Bravo et al., 2008). However, many physical, chemical, and biological factors are involved in bloom development, persistence, and termination (Garcés and Camp, 2012). While most studies on HAB dynamics have focused on bottom-up factors, recent investigations have demonstrated a role for the top-down control exerted by biotic factors, such as parasitism and grazing (Coats et al., 1996; Johansson and Coats, 2002; Calbet et al., 2003; Chambouvet et al., 2008; Montagnes et al., 2008).

Parasitism on marine dinoflagellates by eukaryotic parasitoids is mainly due to members of the globally distributed genera *Parvilucifera* and *Amoebophrya* (Alveolata) (Park et al., 2004). The life cycle of these two parasitoids is, for the most part, similar. Infection begins when a free-living zoospore penetrates a host cell, where it develops into a trophocyte that enlarges while feeding on the host by completely digesting its cell contents. The trophocyte nucleus then divides, and transforms into a sporocyte to produce zoospores. Finally, the newly produced zoospores are released into the marine environment to find a new host (Jephcott et al., 2016). However, whereas after infection by *Parvilucifera* the host stops swimming and sinks immediately (Alacid et al., 2015), dinoflagellates parasitized by *Amoebophrya* continue swimming until late stages of the infection cycle (Park et al., 2002). Moreover, the sporangium of *Parvilucifera* is non-motile, unlike *Amoebophrya*, which forms a highly motile vermiform stage before spreading its zoospores (Fritz and Nass, 1992). Consistent with the features of their infection cycle, marine environmental molecular surveys have highlighted the high occurrence of Marine Alveolates Group II in the planktonic fraction (Guillou et al., 2008; de Vargas et al., 2015; Massana et al., 2015) pointing that *Amoebophryidae* species are much more abundant in the water column than in marine sediment. By contrast, *Parvilucifera* (Perkinsozoa) species are mostly detected in the marine sediment, with a much lower abundance in the water column (Chambouvet et al., 2014). Nonetheless, both groups of parasitoids infect and kill several genera of dinoflagellates, including noxious species, and their very strong virulence and high prevalence have been demonstrated in laboratory experiments and in the field (Coats and Park, 2002; Chambouvet et al., 2008). Thus, some authors have proposed the use of parasitoids as biological control agents for bloom mitigation (Norén et al., 1999; Erard-Le Denn et al., 2000). However, little is known about the specificity of these parasites or the potential unintended side effects on other dinoflagellate populations (Anderson, 2009). The mechanisms underlying host specificity are also not well understood, as intra- and inter-species variability may depend on host phylogeny (Chambouvet et al., 2008) and/or the specific genetic features of the host and parasite. Both will determine the outcome of infection (Råberg et al., 2014; Turon et al., 2015; Alacid et al., 2016).

A few studies have addressed the interaction between parasites and their dinoflagellate hosts in the marine environment in order to assess the impact of parasitism in natural communities. Modeling studies have shown that, under certain conditions, parasitism may have a greater impact than grazing with respect to dinoflagellate population dynamics (Montagnes et al., 2008; Salomon and Stolte, 2010; Jordi et al., 2015). In the field, the prevalence of *Amoebophyra* parasites in dinoflagellate blooms is usually moderate to high (Coats et al., 1996; Chambouvet et al., 2008; Alves-de-Souza et al., 2012; Velo-Suárez et al., 2013) but under some conditions is the main cause of dinoflagellate mortality. In the case of *Parvilucifera*, although its high abundance has been correlated with a reduction in the relative abundance of *A. minutum* in short-lasting blooms (Blanquart et al., 2016), field studies on the prevalence of *Parvilucifera* infections, their impact on natural host populations, and the contribution of infection to bloom termination are lacking.

The main goals of this study were: (i) to determine the timing of *Parvilucifera* parasitoid occurrence in Arenys de Mar harbor, a confined area in the NW Mediterranean Sea; (ii) to assess host-parasitoid dynamics during a bloom of the toxic dinoflagellate *A. minutum*; and (iii) to quantify the impact and contribution of *Parvilucifera* spp. parasitism to bloom termination. The present work constitutes the first record of the impact of *Parvilucifera* parasitoids in the field.

## Materials and methods

### Study area

**Figure 1:**
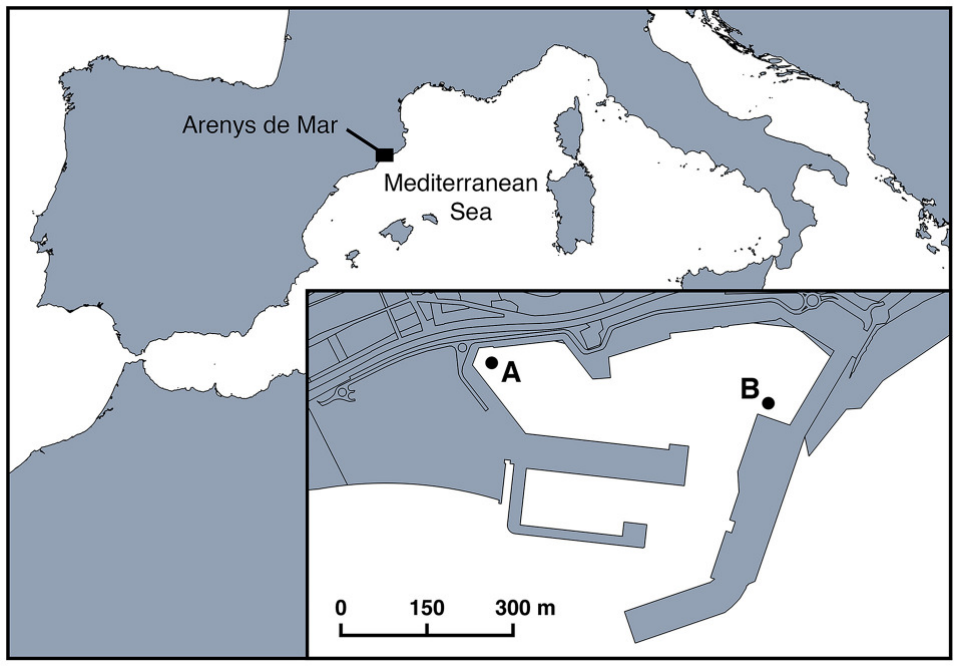
Location of Arenys de Mar harbor in the northwest Mediterranean Sea. The two sampling stations (A and B) are shown in the inset.

Arenys de Mar harbor (41° 34.30′N and 2° 32.40′E) is located on the coast of Catalonia (NE Spain), in the NW Mediterranean Sea (Figure 1). Fishing and leisure are the main human activities in the harbor. The harbor measures 0.4 km^2^, has a depth ranging from 1 m at confined sites to 6 m at the entrance, and receives large freshwater inputs rich in nutrients. Intense and recurrent *A. minutum* blooms between December and August have been recorded every year since 1999 (Vila et al., 2001; Garcés et al., 2004; Van Lenning et al., 2007; Bravo et al., 2008; Anglès et al., 2012). Their documentation was part of an extensive study of the ecology and bloom dynamics of *A. minutum* in this confined system (Garcés et al., 2004; Van Lenning et al., 2007; Anglès et al., 2010, 2012). In the present study, we assessed parasitic occurrence and infection during *A. minutum* blooms at two sampling locations (A and B, Figure 1) where both vegetative cells and resting cysts reached their maximum abundances (Garcés et al., 2004; Anglès et al., 2010).

### Sampling and determination of the dinoflagellate community

Surface water samples were collected at ~20 cm depth using a bucket. From 2009 to 2012, samples were collected from location A once a week between January and September, and once a fortnight from October to December. From January to April 2013, samples were collected at 12:00 GMT at locations A and B (Figure 1) every 5–7 days throughout the *A. minutum* bloom period. This sampling frequency is similar to that followed in previous studies assessing the changes in *A. minutum* abundances during recurrent blooms in the same harbor (Van Lenning et al., 2007; Anglès et al., 2012). To quantify the abundances of the main dinoflagellate species and the standing stock of dinoflagellate hosts, 10- or 50-mL subsamples were fixed in Lugol (2%) and allowed to sediment in settling chambers for 24 h. A Leica-Leitz DMIRB inverted microscope fitted with epifluorescence filters was then used to count at least 400 cells at 20x magnification. The confidence limit at the 95% significance level was ± 10%, and the detection limit using the Utermöhl method 200 cells L^−1^ and 20 cells L^−1^ for the 10-mL and 50-mL samples, respectively (Edler and Elbrächter, 2010). *A. minutum* cells were evidenced by staining the thecal plates with Calcofluor white solution (Fritz and Triemer, 1985).

### Occurrence of *Parvilucifera* parasitoids

*Parvilucifera* occurrence, defined here as the detection of infected cells caused by *Parvilucifera* parasitoids in the samples, was assessed using data obtained from 2009 to 2012 in Arenys de Mar harbor. Thus, 6-L surface water samples were collected with the same sampling frequency used to obtain the plankton samples (Section Sampling and Determination of the Dinoflagellate Community), pre-filtered through a mesh with a 60-μm pore size to discard possible predators, and then incubated for 4–5 days at room temperature (20°C) under natural light. The sample was concentrated by the inverse filtration (10-μm pore size) of a 4-L water subsample and the presence of infected cells, defined as the detection of sporangia, was determined using light microscopy. The detection of <10 and >10 sporangia per concentrated sample was defined as the low and high presence of *Parvilucifera* spp., respectively. Samples with no sporangia were considered to be non-infected.

### Identification and quantification of *Parvilucifera* infections

The impact of *Parvilucifera* infection during a bloom of *A. minutum* was quantified using only the data obtained during the bloom of 2013. Because the dinoflagellate host sinks after *Parvilucifera* infection (Alacid et al., 2015; Turon et al., 2015), two sediment traps were deployed to estimate both the number of infections caused by *Parvilucifera* and the flux of infected cells at locations A and B (Figure 1). Each trap consisted of a cylindrical collection vessel (height 33 cm, diameter 10 cm) moored 0.5 m from the bottom (the depth at each station was 2 m). These traps were similar to those previously employed by Anglès et al. (2012) to quantify the encystment flux of a natural population of *A. minutum*. Under culture conditions, the *P. sinerae* infection cycle in *A. minutum* lasts 3–4 days at a water temperature of 20°C (Alacid et al., 2015) and 5–6 days at a water temperature of 15°C (Råberg et al., 2014). The generation time of *Parvilucifera* at 14°C (average water temperature during the sampling period) was 6–7 days and was estimated using the Q10 temperature corrector (see Section Parasitoid Infection Flux and Prevalence). Based on this estimation, samples were collected from the sediment traps with a frequency of 5–7 days throughout the 2013 bloom period (from January to April). The same sampling frequency was used for host sampling (surface water samples). All settled material was fixed with formaldehyde (1% final concentration) and stored for 1 h in the dark at 4°C. Subsamples (100 mL) were filtered through a mesh of 10-μm, rinsed with 250 mL of autoclaved seawater to remove small sediment particles, and then concentrated in 10 mL of autoclaved seawater in 15-mL Falcon tubes (BD Falcon). From the latter, 1–5 mL were filtered onto 0.8-μm polycarbonate filters (25-mm diameter) using a vacuum pump at 150 mbar at room temperature. Cellulose-acetate support filters were used during filtration to promote the homogeneous distribution of the cells. The filters were cut into pieces with a razor blade, dipped in low-gelling-point agarose (0.1%) to avoid cell loss, and then dried face-down on Parafilm. The filter sections were then mounted on a microscope slide, placed in a mixture consisting of four parts Citifluor and one part Vecta Shield containing 4′-6′-diamidino-2-phelylindole (DAPI; final concentration 1 μg mL^−1^), and stored at 4°C in the dark until they were observed at 400x using an Olympus BX61 epifluorescence microscope. Ultraviolet excitation allowed the detection of the DAPI signal of the host nuclei and of the parasitoid nuclei in the sporangium stage. Blue-light excitation was used to detect the green autofluorescence of *Parvilucifera* parasitoids in the sporangium stage vs. the red autofluorescence of host chlorophyll. *Parvilucifera* infections were confirmed within the whole dinoflagellate community. Parasitoid sporangia were classified into morphotypes based on the size and disposition of the nuclei inside the sporangia and on sporangial morphology. Micrographs were taken using an Olympus DP72 camera (Olympus America Inc.) attached to the microscope. *Parvilucifera* sporangia were counted in 3–4 transects (~11 × 0.5 mm^2^ each) across the filter piece to analyze a representative area of the whole filter. The detection limit under these conditions was ~75 cells L^−1^.

### Parasitoid infection flux and prevalence

The abundance of total parasitoid infections in the sediment traps (data from 2013) was used to determine the daily infection fluxes (infected cells/m^2^ day^−1^) during the bloom. Infection fluxes were obtained by multiplying the abundance of infected cells (infected cells L^−1^) by the trap volume (L^−1^) and dividing first by the trap aperture (cm^2^) and then by the corresponding time interval (days) between the deployment and removal of the traps. To estimate parasitoid prevalence within the host population, the number of infected cells accumulated during the deployment of the sediment traps was divided by the corresponding duration of their deployment to obtain the number of infected cells per day. Parasitoid prevalence within an *A. minutum* population was calculated as the number of infected cells per day as a percentage of the total *A. minutum* population according to:

$$\begin{array}{l} \text{Prevalence}=\frac{\text{Infected cells }\cdot \text{ da}{\text{y}}^{-1}}{A.minutum\text{ standing stock}+\text{ infected cells }\cdot \text{ da}{\text{y}}^{-1}}\times 100 \end{array}$$

Prevalence was similarly determined with respect to the total dinoflagellate community:

$$\begin{array}{l} \text{Prevalence}=\frac{\text{Infected cells }\cdot \text{ da}{\text{y}}^{-1}}{\text{Dinoflagellate standing stock}+\text{infected cells}\cdot \text{da}{\text{y}}^{-1}}\;\times 100 \end{array}$$

This was done to compare the effect of these parasitoids on the blooming host population and on the total dinoflagellate community, since under laboratory conditions *Parvilucifera* species infect a wide range of dinoflagellate species (Figueroa et al., 2008; Garcés et al., 2013a; Lepelletier et al., 2014), reflecting their large number of potential hosts in the field.

### Host mortality due to parasitism

Host mortality induced by *Parvilucifera* parasitoids, i.e., the percentage of hosts killed per day, was estimated as described by Coats and Bockstahler (1994) for *Amoebophrya* parasitoids:

$$\begin{array}{l} \text{Host mortality}=\frac{\left[\frac{\text{ Prevalence}}{\text{ gt}}\right]}{100} \end{array}$$

where gt is the generation time of *Parvilucifera* from the sporangium stage until zoospore release. Here, this value was estimated to be 1.6 days, based on the gt determined for *Parvilucifera sinerae* at 20°C by Alacid et al. (2015) and at 15°C by Råberg et al. (2014) under culture conditions (3.5 and 5.5 days, respectively). The gt was then converted to per day rates of 0.28 and 0.18 day^−1^, respectively. The temperature was corrected by applying the Q_10_ temperature coefficient of 2.5 units, considering the average ambient temperature (14°C) recorded during the *A. minutum* bloom in 2013, and the ~24 h needed for sporangia to release their zoospores at 20°C (Turon et al., 2015). The Q_10_ is a measure of the rate of change of a biological system as a function of an increase in temperature of 10°C. It is estimated by the equation:

$$\begin{array}{l} Q_{10}={\left(\frac{R_{2}}{R_{1}}\right)}^{\left(\frac{10}{T_{2}-T_{1}}\right)} \end{array}$$

where *R* is the per day rate (of change) at a specific temperature (*T*); i.e., *R*_1_ is the per day rate at temperature *T*_1_, and *R*_2_ the per day rate at temperature *T*_2_.

To estimate the contribution of parasitoid-induced mortality to bloom termination, the *in situ* net mortality rate of *A. minutum* was calculated as the decrease in host cell abundance during the decay phase, in this case from March 26th to April 23rd, following the method of Guillard (1973):

$$\begin{array}{l} \mu =\frac{1}{\left(t_{2}-t_{1}\right)}\ln\;\frac{N_{2}}{N_{1}} \end{array}$$

where μ is the mortality rate in days^−1^ and *N*_2_ and *N*_1_ are the cell abundances at *t*_2_ and *t*_1_, respectively. The contribution of parasitoid infection to bloom termination was estimated based on the mean percentage of *A. minutum* and the mortality caused by *Parvilucifera* spp. during the decaying phase of the bloom.

## Results

### Seasonal patterns of *Alexandrium minutum* blooms and parasitoid occurrence during 4 consecutive years

**Figure 2:**
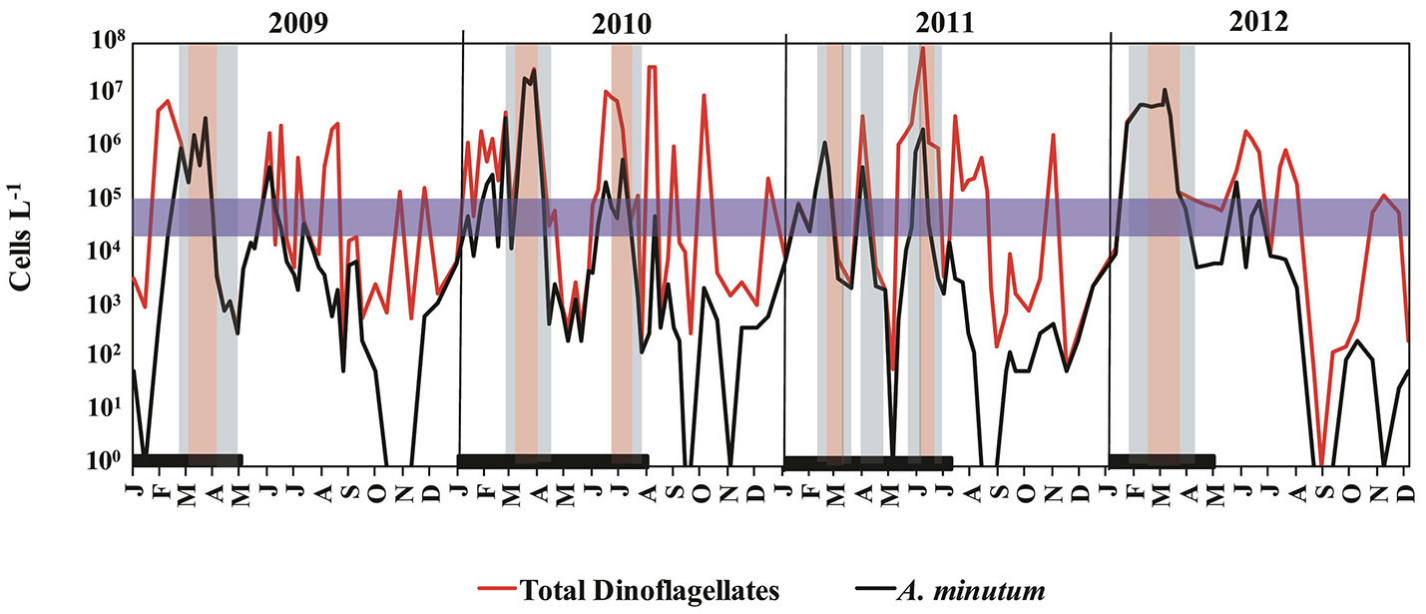
Seasonal patterns of total dinoflagellates (in red), *Alexandrium minutum* abundance (in black), and parasitoid occurrence during a 4-year period (2009–2012) in Arenys de Mar harbor. The thick black line along the bottom indicates the sampling period for *Parvilucifera* parasitoids. The blue horizontal band is the concentration threshold (10^4^–10^5^ cells L^−1^) of *A. minutum* needed to enhance *Parvilucifera* spp. infection. Blue and red shading indicates the low and high presence of *Parvilucifera* spp., respectively.

Between 2009 and 2012, dinoflagellates caused annually recurrent high-biomass blooms at location A. Between June 2010 and 2011, host concentrations at this site were as high as 10^7^ cells L^−1^ (Figure 2). A recurrent peak in *A. minutum* abundance was consistently detected in winter (late February–beginning of March) and caused high-biomass blooms. Other peaks in the abundance of this species occurred in June and July, although the abundances were lower than those reached in winter.

During the sampling period, *Parvilucifera* parasitoids were detected every year, from 2009 to 2012, when *A. minutum* reached bloom abundances as high as 10^4^–10^5^ cells L^−1^ (Figure 2). Parasitic infections in the surface waters were first detected when *A. minutum* abundances were ~10^5^ cells L^−1^ (blue horizontal band in Figure 2). Thereafter, parasite occurrence continued, even when *A. minutum* abundance declined to 10^2^ cells L^−1^. In general, total *Parvilucifera* spp. occurrences lasted 1–2 months, depending on the duration of the specific bloom. In the first weeks of the bloom, the parasitoids had a low-level presence, but when high host abundances were maintained over 2–3 weeks their strong presence was observed, including during the initial phase of the bloom decrease. Finally, at an advanced phase of bloom termination, the parasitoids again reached low abundances, which continued to decline until *Parvilucifera* spp. were no longer detected in the incubated surface water samples.

### Dinoflagellate abundance and composition, infection flux, and parasitoid prevalence during the 2013 winter bloom

**Figure 3:**
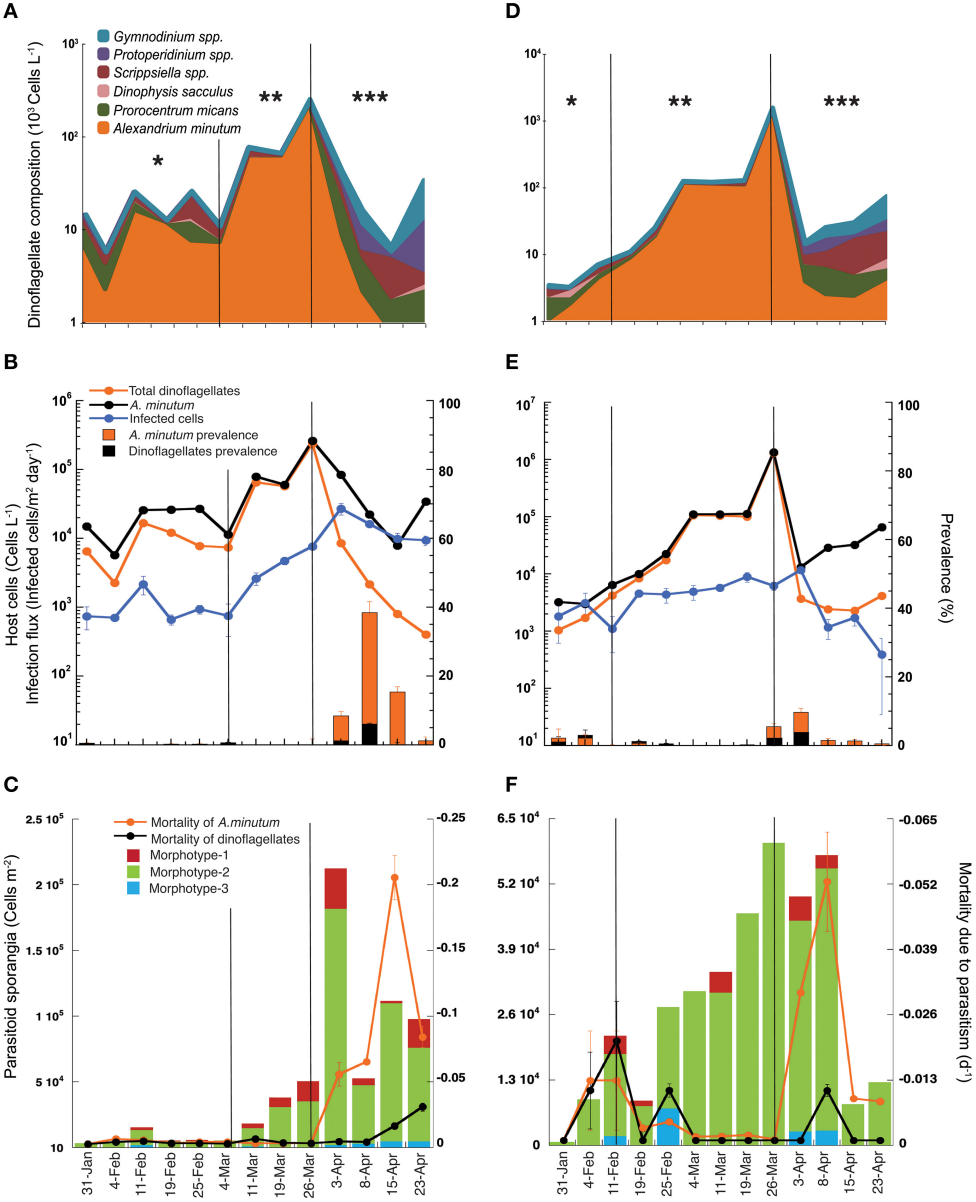
Host-parasitoid dynamics during the winter bloom (from February to April 2013) in Arenys de Mar harbor at locations A **(A–C)** and B **(D–F)**. **(A,D)** Major species composition and contribution to the total dinoflagellate abundance. Note the log scale. **(B,E)** Infection dynamics and prevalence during the bloom. The left-*y* axis is the infection flux (infected cells/m^2^ day^−1^) and host cell abundance (cells L^−1^) of the standing stock. The right-*y* axis is the prevalence (% of infected cells of the population). **(C,F)** The left-*y* axis is the contribution of the different *Parvilucifera* spp. sporangia morphotypes to the total parasitoid density (cells m^−2^), and the right-*y* axis host mortality (day^−1^) caused by the parasitic infection. Asterisks indicate the bloom phase: (^*^) initial phase, (^**^) exponential growth, and (^***^) bloom decrease.

During the sampling period, from February to April 2013 and at both sampling sites, total dinoflagellate abundance increased two-fold from January to late March, reaching a peak on March 26th of up to 10^6^ cells L^−1^ and decreasing to as low as 10^3^ cells L^−1^ in early April (Figures 3A,D). Total dinoflagellate abundance coincided with the fluctuations of *A. minutum*, which was the dominant species during almost the whole sampling period. The bloom of this species lasted 2 months, from early February to early April. During this period, the dinoflagellate species composition changed depending on the bloom phase. Thus, initially, the dinoflagellate community was more diverse but composed principally of *A. minutum, Prorocentrum micans*, and *Scrippsiella* spp. Thereafter, *A. minutum* grew exponentially, with a several-fold increase in its abundance until it dominated the dinoflagellate community (up to 90% of the species contribution). Exponential growth stopped when *A. minutum* reached a peak abundance of up to 10^5^ cells L^−1^ at location A and 10^6^ cells L^−1^ at location B. Abundances of 10^5^ cells L^−1^ were sustained for 10 days at location A and for 15 days at location B; in the latter, abundances increased to as high as 10^6^ cells L^−1^ during a 1-week period. After the peak of the bloom, during the decline in *A. minutum*, the dinoflagellate community again became more diverse, with an increased dominance of *Scrippsiella* spp. and *P. micans*, whereas *A. minutum* abundances reached their lowest values.

*Parvilucifera* spp. infections were restricted to dinoflagellates, but from a total of 17 recorded dinoflagellate taxa (data not shown) only three of them were infected: *A. minutum, Scrippsiella* spp., and *P. micans*. However, while infected cells of *A. minutum* were observed throughout the bloom at both locations, infection of only a few cells of *Scrippsiella* spp. was observed at location A on only two sampling dates, February 19th and March 4th, and of only a single infected cell of *P. micans* on March 4th, also at location A. The detection of infected *Scrippsiella* spp. cells coincided with the maximum abundance of this dinoflagellate (10^4^ cells L^−1^) and with a decrease of *A. minutum*.

The infection flux caused by *Parvilucifera* spp. parasites followed dynamics similar to those of their hosts but with a one-period phase lag between *A. minutum* and *Parvilucifera* densities (Figures 3B,E). The number of infected cells increased gradually at both locations, albeit with a delay, and pointed to a density-dependent response to the increase in host cells during the bloom period. At location A (Figure 3B), during the initial phase of the bloom, host cell abundance in the water column was relatively stable, with values of ~10^4^ cells L^−1^, until March 11th. During this period, the infection flux was also stable, with *Parvilucifera* spp. parasitoids infecting ~10^3^ cells/m^2^ day^−1^. After March 11th, corresponding to the exponential growth phase of the bloom, the infection flux increased one-fold, reaching a maximum on April 3rd of up to 10^4^ infected cells/m^2^ day^−1^. This maximum occurred immediately after the peak in the *A. minutum* concentration (10^5^ cells L^−1^) on March 26th. After the peak, the abundance of A. *minutum* gradually decreased, to 10^3^ cells L^−1^, although the total dinoflagellate concentration was 10^4^ cells L^−1^. The slight decrease in the infection flux coincided with the decrease in *A. minutum* abundance. At location B (Figure 3E), the initial phase of the bloom was much shorter, with the exponential phase starting on February 11th. The infection flux during this initial period was about 10^3^ cells/m^2^ day^−1^, and it slightly increased until a maximum of 10^4^ cells/m^2^ day^−1^ was reached, on April 3rd. The *A. minutum* standing stock of vegetative cells at location B was one order of magnitude higher than that at location A, although the maximum infection flux (10^4^ cells/m^2^ day^−1^), achieved immediately after the peak of the bloom, was the same at the two sites. Following the dramatic decline of the *A. minutum* population, by more than two orders of magnitude, the infection flux declined by one order of magnitude.

The parasitoid prevalences at locations A and B followed a similar pattern (Figures 3B,E), with very low percentages (0–5%) of the host population infected either before peak bloom development during the initial phase or during the exponential growth phase. The percentage of infected hosts increased after the bloom peak, with a mean of 18% at location A and 6% at location B and coinciding at both sites with the rapid decrease in *A. minutum*. Maximum prevalences, reached between April 3rd and 15th, were much higher at location A than at location B, evidenced by maximum values of 38 and 12% of the *A. minutum* population, respectively. The impact of the parasite prevalence on the total dinoflagellate community was very low at both locations over the entire course of the bloom, with a maximum of 7% at location A and 4% at location B.

### Parasitoid sporangial abundance and dinoflagellate mortality due to parasitic infection

Total sporangial abundance followed the same dynamics as the *A. minutum* density, with a higher abundance occurring close to the *A. minutum* (host) peak and then diminishing as the bloom declined (Figures 3C,F). At the initial phase of the bloom, sporangial abundance was slightly lower than during the exponential growth phase, ranging from 10^2^ to 10^4^ cells m^−2^ in the sediment traps. The mean sporangial abundance was 2.5·10^3^ cells m^−2^ at location A and 4.5·10^3^ cells m^−2^ at location B. After the maximum of *A. minutum* abundance, the sporangial density at location A (Figure 3C) increased by one order of magnitude, reaching 2·10^5^ cells m^−2^. The concentration of sporangia decreased only marginally thereafter and was thus maintained at ~5·10^4^–10^5^ cells m^−2^ during the decay phase of the bloom. At location B (Figure 3F), the maximum sporangial density in the traps was 6·10^4^ cells m^−2^, coinciding with the peak in *A. minutum* abundance but then continuing for 2 weeks. This density was one order of magnitude lower than that in location A, although the host cell concentration was higher. Sporangial abundance underwent a sharp decrease when the *A. minutum* abundance declined to <10^3^ cells L^−1^.

**Figure 4:**
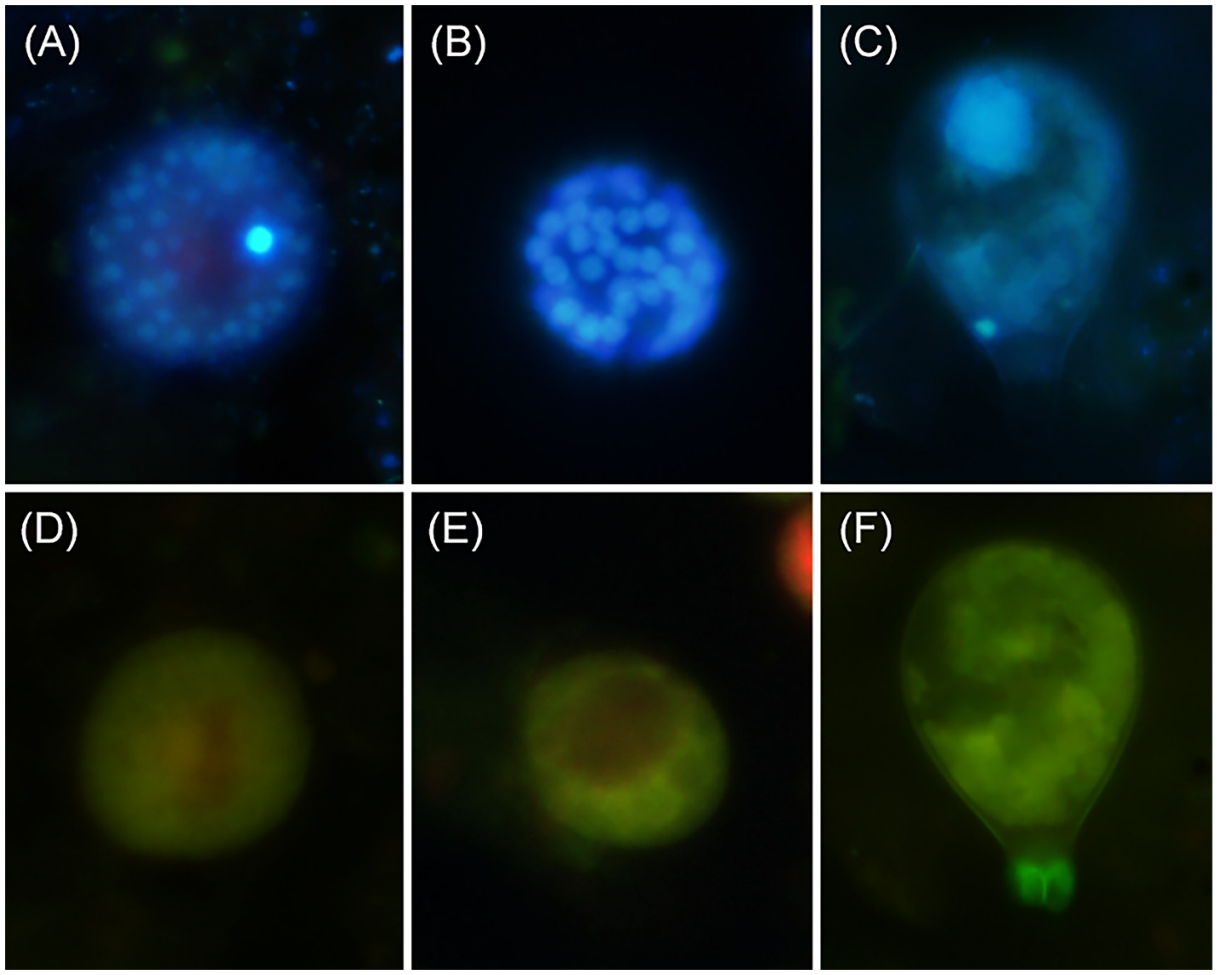
*Parvilucifera* spp. morphotypes identified in the sediment traps during the *A. minutum* winter bloom of 2013. **(A–C)** Sporangia and their DAPI-stained nucleus/nuclei. **(D–F)** The green autofluorescence of the sporangia under blue light excitation. **(A,D)** Morphotype-1; **(B,E)** morphotype-2; **(C,F)** morphotype-3.

Three different *Parvilucifera* sporangial morphologies were identified in samples from sediment traps placed during the *A. minutum* bloom. They could be distinguished based on their nuclear distribution and sporangial morphology (Figure 4). While we were unable to attribute morphotype-1 to any of the five existent *Parvilucifera* species described to date, morphotype-2 was linked to *P. sinerae*, based on the morphological similarities of the sporangia, determined under optical and epifluorescence microscopy, to those described in Alacid et al. (2015). Morphotype-3 was likely related to *Parvilucifera prorocentri*, due to its pear-shaped sporangial morphology (Leander and Hoppenrath, 2008). Morphotype-1 was the second most abundant parasitoid, with a density ranging from 10^2^ to 10^4^ cells m^−2^. This morphotype was present in almost all samples obtained throughout the bloom. Morphotype-2 was the most abundant in the sediment traps and was the cause of most of the infections. In addition, it was dominant during the whole bloom and at both locations (green bar, Figures 3C,F). At location A (Figure 3C), the density of morphotype-2 ranged from 10^3^ to 10^5^ cells m^−2^. Morphotype-3 was only detected sporadically, before the peak of the bloom, and at low concentrations (10^2^ cells m^−2^). It was recurrently present at the late phase of the bloom, from April 3rd to 23rd and at a higher concentration (~10^3^ cells m^−2^). At location B (Figure 3F), the density of morphotype-2 was stable throughout the bloom period at ~10^4^ cells m^−2^, with the lowest abundances at the very early phase and again at the late phase. The other two morphotypes were intermittently detected over the course of the bloom at abundances of ~10^3^ cells m^−2^.

The highest *A. minutum* mortalities due to *Parvilucifera* parasitism occurred after the maximum parasite density (Figures 3C,F) and after the peak host concentration, coinciding with the bloom decay phase at both locations. Before the peaks of host and parasite abundance, *Parvilucifera* spp. killed an average of 0.2% (−0.002 day^−1^) and 0.7% (−0.007day^−1^) of the host population per day at locations A and B, respectively. Host mortality increased after the peak of *Parvilucifera* abundance, with the parasites killing, on average, 10% (−0.1 day^−1^) of the *A. minutum* population every day. The estimated mortality rate during the termination phase of the bloom was lower at location B than at location A, with 2.3% (−0.023 day^−1^) of the host population killed. Maximum host mortalities of −0.21 day^−1^ and −0.053 day^−1^ at locations A and B, respectively, were reached immediately after the maximum in *Parvilucifera* abundances. On average, at location A, *Parvilucifera* parasites killed 3.4% of the *A. minutum* population (−0.034 day^−1^) each day. Considering a decrease of −0.2 day^−1^ in *A. minutum* abundance at the end of the bloom, parasitism due to *Parvilucifera* was estimated to account for 18% of the total *A. minutum* mortality at location A between March 26th and April 23rd, and for 5% at location B during the same period.

## Discussion

### Coupled host-parasitoid occurrence and dynamics in the field

*A. minutum* is a potentially toxic dinoflagellate and a common species in the Mediterranean. In the NW Mediterranean, it is present at low abundances throughout the year in the studied location, but once or twice per year, mainly in winter, it proliferates to form high-biomass blooms. As demonstrated in this study, *Parvilucifera* infections accompany these outbreaks, becoming prominent when the dinoflagellate community is almost mono-specific (>90%) for *A. minutum*. Algal blooms, with their low species diversity and very high abundances (Maso and Garcés, 2006; Sunda et al., 2006; Egerton et al., 2014), are temporary states of the dinoflagellate community. According to the diversity-disease hypothesis of Elton (1958), these communities are vulnerable to parasitic infection and transmission. Elton observed that infectious disease outbreaks due to parasitism most often involve dense, human-simplified communities, such as cultivated land, or, using the example of more recent cultivation systems, marine farmed species (Lafferty et al., 2015). *A. minutum* blooms reported here, with their low species diversity and high cell densities (Figures 3A,D), resemble these systems and thus also support parasitic occurrences. The high host densities reached during dinoflagellate blooms will increase the likelihood of encounter between parasitoids and their hosts (Dobson, 1990), thereby increasing infection transmission and parasitoid load to the system. By contrast, in the absence of a bloom, the low host densities reduce the probability of encounter with susceptible hosts and the infection accordingly subsides.

Infections by *Parvilucifera* parasites at the surface waters were detected only after a peak in *A. minutum* abundance, suggesting the existence of a density threshold that promotes the transmission and spread of *Parvilucifera* infections within the host population. In this study, the minimum *A. minutum* concentration needed to enhance parasitoid infection was ~10^4^–10^5^ host cells L^−1^. Blanquart et al. (2016) followed *A. minutum* and *Parvilucifera* dynamics in two estuaries in France. Whereas, qPCR failed to detect the parasite in the water column at the beginning of the blooms, *Parvilucifera* parasitoids were detected when the density of *A. minutum*, the major contributor to the dinoflagellate community, reached 10^5^ cells L^−1^. The need for a minimum host density that enhances parasite transmission and furthers infections demonstrates a direct host density dependence, in agreement with another density-dependent response described by Garcés et al. (2013b) for the same host-parasitoid system: sporangial activation from a dormant stage. In that process, higher host densities release high concentrations of dimethylsulfide (DMS), which through chemical signaling activates a higher proportion of sporangia containing dormant infective zoospores. DMS “informs” the parasitoid of the presence of a high host abundance in the environment and thus facilitates infection and transmission within the dense host population.

Moreover, as the bloom advances, the increasing host density provokes an increase in the number of infections, thereby enlarging the *Parvilucifera* population. Interestingly, parasitoid abundance was not in phase with the abundance of *A. minutum*; rather, *Parvilucifera* spp. followed a time-delay response to the temporal fluctuations of its blooming hosts, similar to the temporal dynamics of predator-prey interactions. The flux of infected cells was characterized by a lag in host abundance such that infection peaked after the host cells had achieved their maximum density. Then, the parasitoid population in the water column decreased as the bloom declined. This finding is consistent with a direct negative density dependence such as also occurs for predator-prey systems (Begon et al., 2005). Comparable dynamics were also observed in the above-described study by Blanquart et al. (2016) and for parasitoids belonging to the *Amoebophryidae* during blooms of several dinoflagellate species in different locations (Coats and Park, 2002; Chambouvet et al., 2008; Alves-de-Souza et al., 2012). Both *Amoebophryidae* and *Parvilucifera* grow inside the host, killing it as an obligate part of their life-cycles. Therefore, the ecology and population dynamics of parasitoids lie somewhere in between those of predators and true infectious parasites, such as bacteria and protozoa (Hassell, 2000).

The parasitoid community during the bloom could be classified into three *Parvilucifera* morphotypes. The dominance and *A. minutum*-coupled dynamics of morphotype-2 (*P. sinerae*) during the bloom agreed with the results reported by Turon et al. (2015), who, based on the 18S rDNA gene, determined that all *Parvilucifera* isolates from *A. minutum* blooms from the Atlantic and Mediterranean coast (most of them in Arenys de Mar harbor) were *P. sinerae*. Although in host-range laboratory experiments using monospecific cultures *Parvilucifera* species were shown to be generalist pathogens of dinoflagellates (Norén et al., 2001; Garcés et al., 2013a; Lepelletier et al., 2014), the persistent occurrence and dominance of *P. sinerae* during *A. minutum* natural blooms indicated a greater specialization of the parasitoid. This field specificity agrees with the preference of *P. sinerae* for *A. minutum* demonstrated in an artificial mixed community (Alacid et al., 2016). A strong *in situ* specialization is known for *Amoebophrya* parasitoids. Chambouvet et al. (2008) reported the coexistence of several *Amoebophrya* clades, with consecutive blooms caused by a different dinoflagellate species and followed by an increase in the abundance of a specific parasitoid clade. Whether *Parvilucifera* species are characterized by the same dynamics as *Amoebophrya* requires further study of the *in situ* specificity of these parasitoids. The results will reveal details regarding their species- and community-level dynamics and the co-evolution of their hosts.

The host density threshold required to trigger an increase of *Parvilucifera* infections and the delayed density-dependent response of the parasitoid to host abundances have relevant sampling implications. Thus, a specific parasitoid will be significantly abundant in the water column only if its host is also abundant. If at the time of sampling, the host is absent or its density is below the threshold for infection, the parasitoid will not be detected. Moreover, studies of the ecology of *Parvilucifera* must also take into account the meroplanktonic life cycle of these parasitoids, which have a documented benthic stage (i.e., the sporangia) and thus alternate between the water column and the sediment. Taken together, our results explain why, in discrete environmental samples, *Parvilucifera* parasitoids are more abundant and active in the marine sediment (Chambouvet et al., 2014) than in the water column, where they have been scarcely detected (de Vargas et al., 2015; Lepère et al., 2015; Massana et al., 2015).

### Parasitism and bloom termination

Historically, studies on why dinoflagellate blooms decline only considered environmental and physico-chemical factors (Margalef, 1978). However, recent studies have identified biological interactions, such as parasitism and grazing, as important factors in bloom dynamics, since the nature of those interactions affect host population densities (Coats et al., 1996; Johansson and Coats, 2002; Calbet et al., 2003; Montagnes et al., 2008; Velo-Suárez et al., 2013; Alves-de-Souza et al., 2015).

To date, the impact of eukaryotic parasitism in dinoflagellate blooms has been studied only in *Amoebophrya* whereas that of the genus *Parvilucifera* in natural populations is unknown, as only data from laboratory experiments are available. The estimated prevalence of *Parvilucifera* during the *A. minutum* natural bloom, as determined in this study, reached a maximum of 38%, which is much lower than demonstrated in laboratory experiments (>90% for clonal strains) (Alacid et al., 2015, 2016). These differences can be attributed to the many factors in the field that are absent from laboratory conditions, including those that cause direct parasitoid losses, such as grazing on the free-living infective zoospores (Johansson and Coats, 2002), and those that reduce infection success, including dinoflagellate vertical migration (Coats and Bockstahler, 1994), host and parasite genetic diversity (Blanquart et al., 2016), and abiotic conditions such as turbulence (Llaveria et al., 2010).

Although, locations A and B were in the same harbor, they differed remarkably with respect to parasitoid prevalence and host mortality, thus demonstrating the high spatial heterogeneity and patchiness of parasitism as also reported for dinoflagellate vegetative cells during blooms (Garcés et al., 2004). Since host density appears to be fundamental to infection and transmission, a higher infection level was expected at location B, where *A. minutum* abundance was one order of magnitude higher, than at location A. However, parasitoid prevalence and host mortality were higher at the latter location, which points to the role played by the differences in the physical and biological factors of the two locations. Location B is situated close to the entrance of the harbor and is probably more exposed and its waters more turbulent. Turbulence is an important factor that reduces parasite infectivity (Llaveria et al., 2010). Conversely, location A is more protected such that its low hydrodynamics and confinement promoted rates of infection and prevalences similar to those of laboratory conditions. Moreover, mean salinity values during the sampling period were much lower in location A (34.2‰) than in location B (37.8‰). Low salinity levels produce the activation of *P. sinerae* zoospores inside the sporangia and their subsequent release to the environment (Figueroa et al., 2008), which also may explain the higher levels of infection reached in location A. Further studies assessing the contributions of abiotic factors to the success of parasitic infection will allow a better understanding of the variable rates of infection observed in natural blooms. As previously suggested for differences in *P. infectans* infectivity along the Swedish coast (Johansson et al., 2006), differences in the community composition of predators may influence infection rates in the field, as demonstrated for a community rich in grazers in preventing high infection levels (Johansson and Coats, 2002; Kagami et al., 2004; Alves-de-Souza et al., 2015). Although we observed ciliates preying upon *Parvilucifera* zoospores in microscopic observations of living natural samples in the laboratory, nothing is known about the biological interactions of *Parvilucifera* parasitoids with non-host organisms present in the plankton community. Indeed, studies on the relationship of *Parvilucifera* with other trophic levels would be a step forward in understanding energy transfer within marine food webs and in ecological modeling.

In the present study, during the whole bloom period *Parvilucifera* killed, on average, a low percentage (1–3%) of the *A. minutum* planktonic population per day. It was only at the end of the bloom, coinciding with the sharp decrease in *A. minutum* abundance, that *Parvilucifera* reached prevalences high enough to cause maximum host mortalities. Similar patterns were observed in field studies of *Amoebophrya* infection dynamics (Alves-de-Souza et al., 2012; Velo-Suárez et al., 2013). In fact, the host mortalities caused by *Amoebophrya* infection may be so extreme that the bloom collapses (Chambouvet et al., 2008; Mazzillo et al., 2011). Based on a 5–18% decrease in the total *A. minutum* population attributable to *Parvilucifera*, it can be concluded that parasitism strongly influences bloom dynamics, as it leads to losses that are of the same order of magnitude as those due to other biological factors, such as encystment (Anglès et al., 2012) and grazing (Calbet et al., 2003). However, it is not the only cause of bloom termination; in fact, density-dependent disease systems were previously shown to be significantly less likely to cause the extinction of a population (Jaffee et al., 1992). In the case of *Parvilucifera* parasitoids and dinoflagellates, since their interaction is host-density dependent, the natural course of the infection will lower the density of the host and thus its contact rate with the parasitoid. Fewer host individuals and lower infection rates will lead to the establishment of a population equilibrium between dinoflagellate cells and their parasitoids.

Our study demonstrates that the dynamics of *Parvilucifera* are well adapted to those of their blooming hosts and therefore that eukaryotic parasitism is an important factor accounting for biological loss during dinoflagellates massive proliferations. In addition, parasitism exhibits both temporal and spatial heterogeneity during high-biomass blooms. Further investigations of the effects of abiotic and biotic factors on the ecology of these parasitoids are needed to understand parasitoid abundance, host interactions, and the link with other trophic levels of the marine food web.

## Author contributions

EA, JC, and EG designed the study. EA, AR, and EG contributed to data acquisition. EA analyzed the results. EA, AR, JC, and EG contributed to interpretation and discussion of the results. EA prepared the manuscript with contributions from all-coauthors.

### Conflict of interest statement

The authors declare that the research was conducted in the absence of any commercial or financial relationships that could be construed as a potential conflict of interest.
